# Dynamically attentive viewport sequence for no-reference quality assessment of omnidirectional images

**DOI:** 10.3389/fnins.2022.1022041

**Published:** 2022-11-23

**Authors:** Yuhong Wang, Hong Li, Qiuping Jiang

**Affiliations:** ^1^School of Information Science and Engineering, Ningbo University, Ningbo, China; ^2^College of Science and Technology, Ningbo University, Ningbo, China

**Keywords:** omnidirectional images, image quality assessment, no-reference, spatiotemporal scene statistics, virtual reality

## Abstract

Omnidirectional images (ODIs) have drawn great attention in virtual reality (VR) due to the capability of providing an immersive experience to users. However, ODIs are usually subject to various quality degradations during different processing stages. Thus, the quality assessment of ODIs is of critical importance to the community of VR. The quality assessment of ODIs is quite different from that of traditional 2D images. Existing IQA methods focus on extracting features from spherical scenes while ignoring the characteristics of actual viewing behavior of humans in continuously browsing an ODI through HMD and failing to characterize the temporal dynamics of the browsing process in terms of the temporal order of viewports. In this article, we resort to the law of gravity to detect the dynamically attentive regions of humans when viewing ODIs. In this article, we propose a novel no-reference (NR) ODI quality evaluation method by making efforts on two aspects including the construction of Dynamically Attentive Viewport Sequence (DAVS) from ODIs and the extraction of Quality-Aware Features (QAFs) from DAVS. The construction of DAVS aims to build a sequence of viewports that are likely to be explored by viewers based on the prediction of visual scanpath when viewers are freely exploring the ODI within the exploration time *via* HMD. A DAVS that contains only global motion can then be obtained by sampling a series of viewports from the ODI along the predicted visual scanpath. The subsequent quality evaluation of ODIs is performed merely based on the DAVS. The extraction of QAFs aims to obtain effective feature representations that are highly discriminative in terms of perceived distortion and visual quality. Finally, we can adopt a regression model to map the extracted QAFs to a single predicted quality score. Experimental results on two datasets demonstrate that the proposed method is able to deliver state-of-the-art performance.

## Introduction

The omnidirectional image (ODI), which records and delivers 360-degree surround information, plays an important role in virtual reality (VR) photography. The brand-new viewing experience enabled by ODIs is substantially different from traditional 2D plane images, as humans are allowed to freely change their viewport to explore the immersive virtual environments through head-mounted display (HMD) (Li et al., 2018; Zhang et al., 2018; Tran et al., 2019; Deng et al., 2021). Due to its capability of providing natural immersions of real-world scenarios, ODIs have attracted lots of attentions from both academia and industry. In the meanwhile, ODIs have been put into widespread use in many practical VR applications (Chen et al., 2019; ISO, 2019; Alain et al., 2020).

The visual quality of ODIs is a researching-worth topic as ODI content with poor visual quality may cause both physical and mental discomforts. Compared with the traditional 2D plane image, the visual quality issues of ODIs are much more acute and challenging. On the one hand, the currently mainstream methods for acquiring a typical ODI are to stitch multiple images which are captured by using a wide-angle camera array with partially overlapped field of view. These images from multiple cameras are stitched to produce an omnidirectional panorama in the spherical format. However, the stitching process will inevitably introduce stitching artifacts at the stitching boundaries, which are scarcely occurred in traditional 2D plane images (Ho and Budagavi, 2017). On the other hand, compared with traditional 2D plane images, the storage space requirement and the spatial resolution of ODIs are much higher, e.g., 4K, 8K, or higher. Therefore, ODIs are often heavily compressed to facilitate transmission and storage (Kim et al., 2020). As a result, ODIs with serious compression artifacts inevitably lead to even worse quality-of-experience. In addition, ODIs are usually with the spherical format to be displayed on HMD. Therefore, the human viewing behavior when freely exploring the ODIs with HMD is dramatically different with that of 2D plane images. This dramatically different viewing behavior will also affect the human quality perception of ODIs accordingly. On considering the above quality issues, it is necessary to develop effective ODI quality metrics by jointly considering the effects of different distortions as well as the human viewing behaviors in viewing ODIs with HMD.

Despite its high importance, the problem of ODI quality evaluation has been not well addressed so far. One of the most important challenges is that human viewing behavior in browsing ODIs through HMD is dramatically different from that in viewing 2D plane images by human eyes directly. Typically, when viewers are browsing a spherical ODI through HMD, they can obtain immersive and interactive viewing experiences by freely changing the viewpoint, and only the visual contents within the current viewport can be viewed at a certain time (Chen et al., 2020; Xu M. et al., 2020). Besides, viewers usually tend to focus on the regions near the equator during the exploration of ODIs with HMD. Therefore, the prediction of viewports plays a critical role in designing accurate ODI quality evaluation metrics as it can extract the most important visual contents from ODIs to facilitate the quality evaluation process. To account for this, some previous efforts have been made to extract viewports from ODIs for ODI quality evaluation. For example, the VGCN-based ODI quality evaluation method proposed in Xu J. et al. (2020) was dedicated to extracting viewports with higher probabilities of being explored by viewers according to the human visual sensitivity to structural information and adopted the graph convolution network (GCN) to predict ODI quality score by implicitly modeling the interactions among different viewports. In Sun et al. (2019), the authors proposed to project the equirectangular image into six equally sized viewport images and then generate a corresponding channel for further study. These previous works have achieved promising performance and demonstrated the validity and importance of viewport generation toward creating reliable ODI quality evaluation metrics. Despite the effectiveness, the viewport generation strategies of these methods have a common limitation, i.e., they both ignore the actual viewing behavior of humans in continuously browsing an ODI through HMD and fail to characterize the temporal dynamics of the browsing process in terms of the temporal order of viewports. Therefore, research efforts dedicated to more accurate viewport sequence generation by accounting for the temporal order of viewports will undoubtedly facilitate the quality evaluation of ODIs.

In this article, we propose a novel no-reference (NR) ODI quality evaluation method by making efforts on two aspects including the construction of Dynamically Attentive Viewport Sequence (DAVS) from ODIs and the extraction of Quality-Aware Features (QAFs) from DAVS. The proposed method is named Spatiotemporal Scene Statistics of Dynamically Attentive Viewport Sequence (S^3^DAVS) in short. The construction of DAVS aims to build a sequence of viewports that are likely to be explored by viewers based on the prediction of visual scanpath when viewers are freely exploring the ODI within the exploration time *via* HMD. A DAVS that contains only global motion can then be obtained by sampling a series of viewports from the ODI along the predicted visual scanpath. As a result, the obtained DAVS can be considered a human viewing behavior-characterized compact representation of the whole ODI. The subsequent quality evaluation of ODIs is performed merely based on the DAVS. The extraction of QAFs aims to obtain effective feature representations that are highly discriminative in terms of perceived distortion and visual quality. Finally, we can adopt a regression model to map the extracted QAFs to a single predicted quality score.

The contributions of this work are 2-fold. First, we make the first attempt to predict the visual scanpath based on which a DAVS is accordingly obtained as one kind of human viewing behavior-characterized compact representation of the whole ODI. Second, we model the spatiotemporal scene statistics by analyzing the 3D-MSCN and spatiotemporal Gabor response maps of the DAVS to serve as the QAFs for the quality evaluation of ODIs. Finally, we conduct extensive experiments on two benchmark databases to validate the effectiveness of our proposed S^3^DAVS method.

The rest of this article is arranged as follows. Section “Related works” provides a brief review of some representative ODI quality metrics. Section “Proposed spatiotemporal scene statistics of dynamically attentive viewport sequence approach” illustrates the details of our proposed ODI quality evaluation metric. Section “Experimental results” presents the experiments and performance comparisons. Section “Conclusion” concludes and discusses future works.

## Related works

Compared with traditional 2D plane images, ODIs are always extremely high-resolution and a large amount of data which hereby directly increases the burden of transmission and storage. Therefore, the visual quality issues of ODIs are much more acute and challenging, calling for dedicated ODI quality evaluation metrics. During the past decades, image quality evaluation has been widely investigated and can be roughly categorized into three categories including full-reference (FR), reduced-reference (RR), and no-reference (NR) according to the participation amount of information from reference image (i.e., without distortion) (Mittal et al., 2013; Zhu et al., 2019; Liu et al., 2021). Compared with the FR/RR methods which require full/partial reference information, the NR methods do not require any information from the reference image, which can have much wider applications in practical systems. In this section, we will provide a brief review of some representative FR and NR ODI quality metrics in the literature.

### FR-OIQA

There have been many FR image quality assessment (IQA) metrics designed for 2D plane images (Wang et al., 2003; Zhou et al., 2004; Sheikh et al., 2006; Zhang et al., 2011; Xue et al., 2014). However, the existing 2D FR-IQA metrics cannot be directly applied to evaluate ODIs because the geometric distortions induced by projection will be wrongly treated and evaluated. Therefore, the previous research efforts mostly concentrate on studying to improve existing 2D FR-IQA metrics by removing the influence of the projection-related geometric distortions. Yu et al. (2015) proposed a spherical PSNR (S-PSNR) metric to improve the performance of traditional PSNR by sampling pixel locations from a sphere uniformly and getting pixel contents from the reference image and the distorted image according to the relation of spherical coordinates to 2D coordinates, then calculated the error between the pixels on the reference image and the pixels on the distorted image. Zakharchenko et al. (2017) introduce a Craster’s parabolic projection (CPP-PSNR) method by projecting the reference image and distorted image into a shared CPP format domain before calculating the PSNR. However, the drawback of CPP-PSNR methods is that interpolation may introduce errors and decrease the accuracy. Sun et al. (2017) advocated calculating weighted-to-spherically uniform PSNR (WS-PSNR) on the 2D format of the omnidirectional image directly, which may decrease the negative effect of interpolation in CPP-PSNR. However, PSNR is not highly correlated with human perception (Horé and Ziou, 2010). Inspired by the good consistency of structure similarity (SSIM) metrics with subjective perception in 2D-IQA, Zhou et al. (2018) introduce a weighted-to-spherically uniform SSIM metric for FR-OIQA. Besides, some studies suggest figuring out more information from other dimensions to improve performance. For example, the phase consistency-guided method (Xia et al., 2021) proposes to utilize the abundant structure and texture features of high-order phase information from ODIs. Recently, with the development of deep learning, Kim et al. (2020) constructed a novel FR-OIQA model by learning the visual and positional features with the guidance of human perception through adversarial learning.

### NR-OIQA

The limitation of FR-OIQA metrics is the dependence on reference images which may be unavailable in practical applications. Therefore, NR-IQA metrics that do not require any reference information are desired (Gu et al., 2015). In the literature, several NR-IQA metrics for 2D images have been proposed (Moorthy and Bovik, 2011; Min et al., 2018; Yan et al., 2019). However, as demonstrated in Xu J. et al. (2020), NR-OIQA involves new challenges, such as stitching artifacts, sphere representation, and wide field of view (FoV). Therefore, a reliable no-reference quality metric for ODIs is extremely needed. There have been some algorithms proposed for NR-IQA of ODIs. Zhou W. et al. (2022) captured the multi-frequency information by decomposing the projected ERP maps into multiple images on sub-bands. However, the conventional method of studying the overall ODI is not necessary, because the salient region in ODIs mainly locate on the equator (Sitzmann et al., 2018). Therefore, the thought of exploring the information from a viewport-based image is proposed and obtained broad acceptance. Sun et al. (2019) projected each ODI into six viewport images and then proposed a multi-channel CNN framework including six parallel ResNet34. However, the way to obtain viewport-based images is directed by the behavior of humans when they view omnidirectional images through HMD (Li et al., 2019; Xu et al., 2019; Jiang et al., 2021; Zou et al., 2021). Xu J. et al. (2020) noticed this problem and constructed a viewport-oriented graph convolutional network (VGCN) to address the perceptual quality assessment of ODIs.

## Proposed spatiotemporal scene statistics of dynamically attentive viewport sequence approach

**FIGURE 1:**
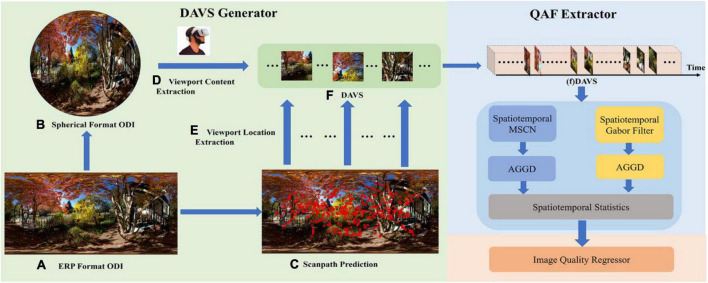
The overall framework of our proposed S^3^DAVS method. It contains DAVS generator, QAF extractor, and image quality regressor. The viewport sequence produced by the DAVS generator will be sent into the QAF extractor and then the image quality regressor will predict the final quality score based on the features coming from the QAF extractor. Source for the photos in this figure: open source OIQA Database.

The framework of our proposed method is shown in Figure 1. Our proposed method involves three modules, including DAVS generator, QAF extractor, and image quality regressor. DAVS which is composed of a series of viewports sampled from the ODI along the scanpath can be considered a human viewing behavior-characterized compact representation of the whole ODI. Specifically, part (a) is an unprocessed ERP image from the OIQA database. The spherical format ODI converted from ERP format ODI is presented in part (b). The predicted results of scanpath are shown in part (c). We get the center location of each viewport on spherical format ODI from precomputed locations in the ERP format ODI provided in part (c) by coordinate mapping. Then, we offer the value of FOV to set the size of the viewport, and finally obtain the complete viewport content from part (b). All those viewport contents are shown in part (f). In the QAF extractor, the image sequence is generated by arranging the viewport images along the order in the scanpath. The QAF extraction is performed on the DAVS and involves obtaining effective feature representations that are highly discriminative in terms of visual quality. Finally, the extracted QAFs are used to predict the quality score *via* a learned regression model.

### Dynamically attentive viewport sequence construction

*1) Scanpath prediction:* Sitzmann et al. (2018) proposed that humans mainly pay attention to salient regions located on the equator in ODIs. Therefore, our original intention of building this omnidirectional image quality assessment algorithm is to simulate human behavior when they observe ODI through HMD (Zhu et al., 2020). Besides, different trajectories of visual scanpath will produce different contents of DAVS and accordingly affect the perceptual quality assessment. Based on these two considerations, the key to DAVS construction is accurately predicting the scanpath of viewers when they are exploring the virtual scene *via* HMD. The fixation is decided by visual attention shift, i.e., a phenomenon of the temporal dynamics of human visual attention (Yang et al., 2021). Current approaches in modeling scanpath prediction are based on the saliency map that illustrates the probability of each human visual attention (Ling et al., 2018). However, these approaches fail to characterize the temporal dynamics of visual attention. Recently, it has been revealed that the law of gravitation can well explain the mechanisms behind the dynamic process of attention shift in exploring a visual scene. Inspired by this, we intuitively propose a gravitational model-based visual scanpath prediction approach for ODIs. Given an ODI image as input, this model can yield a continuous function that describes the trajectory of the fixations over the exploration time as output.

We think the process that humans pay attention to the interesting area can be regarded as a field effect in HMD. Therefore, it is necessary to define the virtual mass corresponding with ODI content and the virtual field in ODIs for building a relationship between ODI content with fixation. Here, we employ the model proposed by Zanca and Gori (2017) and Zanca et al. (2020) for the utilization of gravitation theory. The ODI content noticed by humans should contain abundant details and motion information. Details reflect masses proportional to the magnitude of the gradient, while motions illustrate proportional to the optical flow which mainly occurs when humans shift to the next fixation. If we set virtual mass μ that obeys a certain distribution as the assembling of all the fixation during the exploration, and think it degenerates to a single distributional mass concentrated in *x*, then the virtual mass can be expressed as μ(*y*,*t*) = δ(*y*−*x*), *y* = *a*(*t*),where *a*(*t*) represents the focus of attention at time *t*. The μ(*y*,*t*) consists of two elements: gradient of brightness μ_*1*_ and optical flow μ_*2*_. The field we set makes effects on the virtual mass we have designed, and then the Green function (*G*) will construct the correlation between the field and its corresponding mass. Therefore, our scanpath can be associated with attention *a*(*t*) by the potential,


(1) $$\begin{array}{c} G\,\left(a\;-\;x\right)=-\frac{1}{2\pi }\,\log\left(||a-x||\right) \end{array}$$

Then the gravitational field *e* at time *t* can be written as:


(2) $$\begin{array}{c} e\;\left(a\;-\;x\right)=\frac{1}{2\pi }\,\frac{a-x}{{||a-x||}^{2}} \end{array}$$

where Equation (2) illustrates that the strength of field is inversely proportional to the distance between the *a*(*t*) and *x*. Then the overall field can be demonstrated by,


(3) $$\begin{array}{c} E\;\left(a\,\left(t\right)\right)=-\frac{1}{2\pi }\,\int_{R}d\,x\,\frac{a\,\left(t\right)-x}{{||a\,\left(t\right)-x||}^{2}}\,\mu \,\left(x,t\right) \end{array}$$

(4) $$\begin{array}{c} E\;\left(a\,\left(t\right)\right)=-\left(e*\mu \right)\;\left(a\,\left(t\right)\right) \end{array}$$

where Equation (3) can be rewritten as Equation (4) using convolution operation. It depicts the links between the virtual mass in the field and the attention of viewers. Subscript *R* represents the overall field. However, the method first explores the focus of attention in the most attentive regions which may hinder the method from searching unexplored locations and finishing a complete exploration of the scene. Therefore, the shift of attention is needed to be triggered by an extra setting. Zanca proposed to introduce the inhibitory function (*I*(*t*)) which will return value 0 for the pixels that have not been explored and value 1 for the pixels that have already been explored. Then all the focus of attention *a*(*t*)can finally be represented as follows:


(5) $$\begin{array}{c} \dot{I}\;\left(t\right)=\beta \,\left(g\;\left(x-a\,\left(t\right)\right)-I\,\left(t\right)\right) \end{array}$$

(6) $$\begin{array}{c} \dot{a}\;\left(t\right)=z\;\left(t\right) \end{array}$$

(7) $$\begin{array}{c} \dot{z}\;\left(t\right)=-\lambda \,z\;\left(t\right)-\left(e*\mu \right)\;\left(t,a\;\left(t\right)\right) \end{array}$$

**FIGURE 2:**
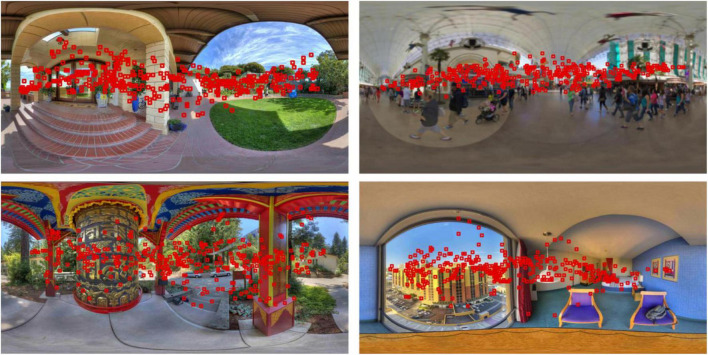
Examples of scanpath prediction results. The red squares in each image denote fixation locations based on which the viewport images are sampled. Source for the photos in this figure: open source OIQA Database.

Here, $g\,\left(u\right)=e^{-\frac{u^{2}}{2\,\delta^{2}}}$ and 0 < β < 1. The dumping term $\dot{a}\,\left(t\right)$ prevents strong oscillations and makes the overall dynamics closer to human scanpath. Figure 2 shows the scanpath prediction of four images in the OIQA database. We could obtain specific coordinates of these viewport centers to be utilized in the following part.

**FIGURE 3:**
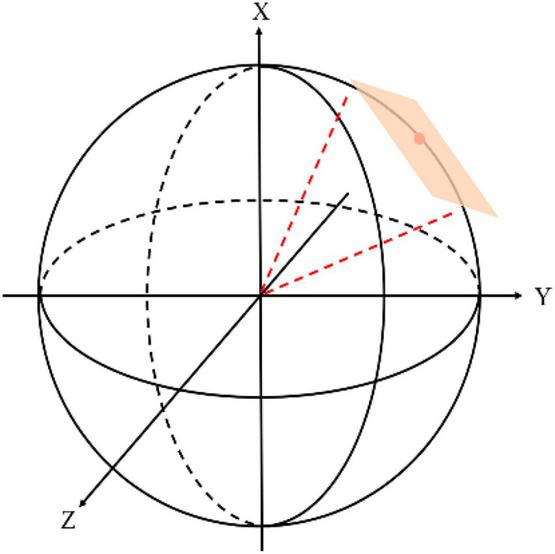
The illustration of the spherical scene. The viewer with HMD stands at the center of the sphere. The location of attention can be described with both longitude and latitude.

**FIGURE 4:**
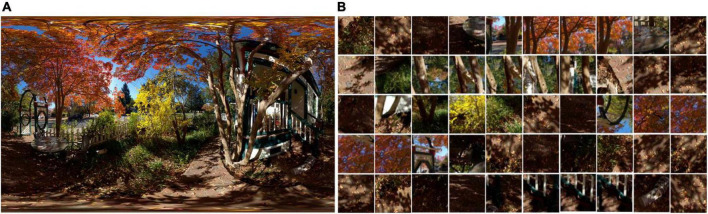
Visualization of viewports in DAVS obtained by the scanpath-based viewport sampling. **(A)** ODI with the ERP format and **(B)** examples of different viewports in DAVS. Source for the photos in this figure: open source OIQA Database.

*2) Scanpath-based Viewport Sampling:* Based on the predicted scanpath data, we can obtain a series of viewport images from the ODI by sampling along the trajectory of the scanpath. The precomputed locations denoted by {*s*_1_,*s*_2_,…,*s*_*t*_} are human fixations that have been listed along the timeline, but they only represent the center of attentive regions on the ERP ODIs. Each *s*_*t*_ includes *s*_*t,x*_ and *s*_*t,y*_, where, (*s*_*t,x*_, *s*_*t,y*_) correspond with width and height values on the ERP image. To obtain the realistic viewport content from the spherical scene, we first map those locations onto a unit sphere and then define those coordinates as the predicted scanpath which is written as {*p*_1_,*p*_2_,…, *p*_*t*_}, where the subscript of *s*_*t*_*andp*_*t*_ records the overall exploration time and keep the consistency between locations from the ERP image and locations from the spherical image. Specifically, given the current attentive location in the ERP image is *s*_*t*_, we calculate its spherical location *p*_*t*_ and consider it to be the actual fixation. Then, based on the theory about near peripheral vision (Besharse and Bok, 2011), we first set the field of view (FoV) to [-π/6, π/6], after which the scale of viewport content can be determined (Sui et al., 2022). Finally, bicubic interpolation will be used to sample the viewport image and construct the sequence according to the order of the time. Figure 3 shows how viewers perceive spherical content with HMD at time *t*. In the spherical coordinate, the behavior of viewer position mainly decided by rotation matrix (Rai et al., 2017) and is mainly calculated by the Cartesian theory for the location transformation. Figure 4 shows partial viewport content from the DAVS. (A) is an original distorted ERP image in the database and (B) displays the examples of different viewports in DAVS. Those overlapping viewport contents illustrate a dynamically attentive process when humans view the ODI from one attentive point to the next and finally composite into a sequence that curve the dynamically spatiotemporal information which will facilitate us to extract spatiotemporal features from the DAVS. Comparing the content in these viewports, some of these viewport contents are rich in luminance information, while others are not. This phenomenon corresponds to the theory of not depending on the saliency map but predicting the scanpath with the law of gravity, so the scanpath is more accurate than the common method.

### Quality-aware feature extraction and quality prediction

As stated, the built DAVS can be considered as a human viewing behavior-characterized compact representation of the whole ODI. The remaining issue is how to perform the quality evaluation of an ODI based on the DAVS. As known, the most important step of quality evaluation is to extract effective QAFs that are highly descriptive of distortion level (i.e., visual quality level). Since the DAVS is a sequence composed of a series of viewports sampled from the ODI along the scanpath, we can naturally treat the DAVS as a pseudo video with only global camera motion and each viewport in DAVS just corresponds to a specific frame in the video. Obviously, just like a natural video, the DAVS also contains information regarding the spatial variations in pixels along with the dynamic motion in successive frames (Mittal et al., 2016). Thus, the QAF extraction from DAVS should well account for the characteristics in the spatiotemporal domain. In this work, we propose to model the spatiotemporal scene statistics of the DAVS as the QAFs of the corresponding ODI. The obtained spatiotemporal scene statistics are used as the QAFs based on which quality prediction is performed.

*1) Spatiotemporal MSCN Sequence:* It has been widely demonstrated that the local mean subtracted contrast normalized (MSCN) coefficients of a pristine natural image can be well modeled by a Gaussian distribution (Mittal et al., 2012). However, when a natural image suffers distortions, the distribution of its MSCN coefficients will deviate from the original distribution (Mittal et al., 2012). These distributions can be modeled either by a generalized Gaussian distribution (GGD) or asymmetric GGD (AGGD). The parameters of GGD and AGGD can be used as the QAFs of natural images and have achieved great success in designing NR-IQA metrics during the past decade. The main reason for working on MSCN images is due to the decorrelation of local pixel dependency. Similarly, the DAVS which records the dynamic visual contents seen by the viewers along the scanpath has a high correlation among neighboring pixels in both spatial and temporal domains. In order to decorrelate such local dependency in the DAVS, we propose to use the spatiotemporal MSCN. Specifically, let us denote the DAVS as *V*(*x*,*y*,*t*), and the spatiotemporal MSCN coefficients of *V*(*x*,*y*,*t*) is calculated as follows:


(8) $$\begin{array}{c} \hat{V}\,\left(x,y,t\right)=\frac{V\,\left(x,y,t\right)-\mu \,\left(x,y,t\right)}{\sigma \,\left(x,y,t\right)+c} \end{array}$$

where *x* and *y* are the spatial indices and *t* is the temporal index. A small constant *c* is imposed on the dominator to avoid instability when σ(*x*,*y*,*t*) approaches to zero and we empirically set *c* = 1 in this work. μ(*x*,*y*,*t*) and σ(*x*,*y*,*t*) are the mean and standard deviation, respectively, which are defined as follows:


(9) $$\begin{array}{c} \mu \;\left(x,y,t\right)=\sum_{j=-J}^{J}\sum_{k=-K}^{K}\sum_{l=-L}^{L}\omega_{j,k,l}\,V\;\left(x+j,y+k,t+l\right) \end{array}$$

(10) $$\sigma \,\left(x,y,t\right)=\sqrt{\sum_{j=-J}^{J}\sum_{k=-K}^{K}\sum_{l=-L}^{L}\omega_{j,k,l}\,{\left[V\,\left(x+j,y+k,t+l\right)-\mu \,\left(x,y,t\right)\right]}^{2}}$$

where ω is a symmetric normalized 3D Gaussian weighting function with zero mean and standard deviation of 1.166. According to Dendi and Channappayya (2020), we set *J* = *K* = *L* = 2.

*2) Spatiotemporal Gabor Filter Response Sequence:* It has been hypothesized that the HVS employs spatiotemporal bandpass filters to analyze and process dynamic visual signals (Simoncelli and Olshausen, 2001). Our proposed approach is motivated by this hypothesis that spatiotemporal Gabor filters are a good approximation of the bandpass behavior of the HVS (Petkov and Subramanian, 2007; Tu et al., 2020; Gotz-Hahn et al., 2021). The spatiotemporal Gabor filters combine information over space and time, which advocates a suitable model for feature analysis of the DAVS. Mathematically, the spatiotemporal Gabor filter is defined by the product of three factors including a Gaussian envelope function that limits the spatial extent, a cosine wave moving with phase speed *v* in the θ direction, and a Gaussian function that determines the decay along time:


(11) $$G_{v,\theta ,\phi }\;\left(x,y,t\right)=\frac{\gamma }{2\,\pi \,\sigma^{2}}\,\exp\left(\frac{-\left({\left(\bar{x}+v_{c}\,t\right)}^{2}+\gamma^{2}\,{\bar{y}}^{2}\right)}{2\,\sigma^{2}}\right)$$

$$\cdot \cos\left(\frac{2\pi }{\lambda }\,\left(\bar{x}+\mathrm{vt}\right)+\varphi \right)\cdot \frac{1}{\sqrt{2\pi }\tau }\,\exp\left(-\frac{{\left(t-\mu_{t}\right)}^{2}}{2\,\tau^{2}}\right)$$

where $\bar{x}=x\,c\,o\,s\,\left(\theta \right)+y\,s\,i\,n\,\left(\theta \right),\bar{y}=-x\,s\,i\,n\,\left(\theta \right)+y\,c\,o\,s\,\left(\theta \right)$. γ is the rate that specifies the ellipticity of the Gaussian envelope in the spatial domain and is set to γ = 0.5 for matching to the elongated receptive field along the $\bar{y}$ axis. σ describes the standard deviation of Gaussian and determines the size of the receptive field. *v* is the phase speed of the cosine factor, which determines the speed of motion. In addition, the speed which the center of the spatial Gaussian moves along the $\bar{x}$ axis is specified by the parameter *v*_*c*_. In our implementation, we simply set *v*_*c*_ = *v*. λ is the wavelength of the cosine wave and it is obtained through the relation $\lambda =2\,\sqrt{1+v^{2}}$. θ ∈ [0,2π] determines the motion direction and the spatial orientation of the filter. The phase offset φ ∈ [−π,π] determines the symmetry in the spatial domain. The Gaussian distribution with mean μ_*t*_ = 1.75 and standard deviation τ = 2.75 is used to model the decay in intensities along time.

With the above-defined spatiotemporal Gabor filter, the bandpass response of a specific spatiotemporal MSCN sequence can be obtained by convolving it with a bank of spatiotemporal Gabor filters:


(12) $$\begin{array}{c} R_{v,\theta ,\phi }\;\left(x,y,t\right)=\hat{V}\;{\left(x,y,t\right)}^{*}\;G_{v,\theta ,\varphi }\;\left(x,y,t\right) \end{array}$$

where * denotes the convolution operation. In this work, we generate the bandpass spatiotemporal Gabor filter banks by varying the values of *v*, θ, and φ. Specifically, we select three different speeds {*v* = 0,1,2}, four different orientations {θ = 0,π/3,2π/3,π}, and two different phase offsets, i.e., φ = 0 for the symmetry case and φ = π/2 for the anti-symmetry case. As a result, the total number of spatiotemporal Gabor filters is 24. It means that we can obtain 24 bandpass spatiotemporal Gabor filter response sequences for an input DAVS.

*3) AGGD Modeling and Parameter Estimation:* In the legacy NR-IQA works, it has been widely demonstrated that the distribution of the coefficients in a 2D MSCN map can be well modeled by GGD or AGGD. Inspired by this, we in this work also employ the AGGD to model the distributions of the coefficients in the spatiotemporal MSCN and bandpass spatiotemporal Gabor filter response sequences, respectively. The AGGD is a flexible model that can effectively characterize a large variety of unimodal data with only three parameters. Mathematically, the AGGD model is described as follows:


(13) $$\begin{array}{c} f\;\left(x;\gamma ,\beta_{l},\beta_{r}\right)=\left\{ \begin{array}{c} \frac{\gamma }{\left(\beta_{l}+\beta_{r}\right)\,\Gamma \,\left(\frac{1}{\gamma }\right)}\,\exp\left(-{\left(\frac{-x}{\beta_{l}}\right)}^{\gamma }\right);\forall x\leq 0\\ \frac{\gamma }{\left(\beta_{l}+\beta_{r}\right)\,\Gamma \,\left(\frac{1}{\gamma }\right)}\,\exp\left(-{\left(\frac{-x}{\beta_{r}}\right)}^{\gamma }\right);\forall x>0 \end{array}\right. \end{array}$$

where γ,β_*l*_,β_*r*_ are three parameters controlling the shape of the distribution, and Γ(⋅) is defined as follows:


(14) $$\begin{array}{c} \Gamma \,\left(a\right)=\int_{0}^{\infty }t^{a-1}\,e^{-t}\,\mathrm{dt};a>0 \end{array}$$

We adopt the moment estimation method suggested in Lasmar et al. (2009) to estimate the three parameters γ,β_*l*_,β_*r*_. Besides, we also compute another parameter η = γ/(β_*l*_ + β_*r*_). Finally, we use four parameters [γ,β_*l*_,β_*r*_,η] to represent an AGGD model.

*4) Final Feature Representation:* By applying the AGGD to model the distributions of the coefficients in the spatiotemporal MSCN and bandpass spatiotemporal Gabor filter response sequences, we can obtain a hybrid parameter set to serve as the QAF representation of the DAVS:


(15) $$\begin{array}{c} {\hat{f}}_{D\,A\,V\,S}=\left[{\hat{f}}_{\hat{V}},{\hat{f}}_{R_{v,\theta ,\varphi }}\right] \end{array}$$

where ${\hat{f}}_{\hat{V}}$ is a 12-dimensional feature vector containing the AGGD parameters obtained by applying the AGGD model on the spatiotemporal MSCN sequence at three coarse-to-fine scales (the coarser scale is first processed by a low-pass filter, followed by a down-sampling operation with a factor of 2 and ${\hat{f}}_{R_{v,\theta ,\varphi }}$ is a 288-dimensional feature vector also containing the AGGD parameters at three coarse-to-fine scales and they are obtained by applying the AGGD model on the bandpass spatiotemporal Gabor filter response sequences with all *v*, θ, and φ.

*5) Quality Score Regression*: After obtaining the overall feature representation of the DAVS (also the ODI), the remaining issue is how to predict the quality score of an input ODI based on the extracted feature representations. Given the subjective quality is provided in the form of scaler, the quality prediction is a typical regression problem from the perspective of machine learning. Therefore, we learn a regression model *via* support vector regression (SVR) for mapping the 300-dimensional QAF vector into a single quality score with the usage of a radial basis function kernel. Once the SVR model is built by training, it can be used for the quality prediction of an input ODI in the test stage with its corresponding 300-dimensional QAF vector as input.

## Experimental results

### Experimental protocols

We utilize two publicly available omnidirectional image quality databases in the experiments. They are the OIQA database (Duan et al., 2018) and CVIQD database (Sun et al., 2018). The OIQA database contains 16 image scenes with four distortion types, including JPEG compression (JPEG), JPEG2000 compression (JP2K), Gaussian blur (BLUR), and Gaussian white noise (WN). Further, each distortion type involves five distortion levels. So, the OIQA database includes 16 pristine ODIs and 320 distorted ODIs in total. Subjective rating scores in the form of mean opinion score (MOS) are given in the range of Xue et al. (2014) and Tran et al. (2019), where a higher score means better visual quality. The CVIQD database consists of 528 ODIs including 16 pristine images and 512 compressed ODIs. Three popular coding techniques, i.e., JPEG, H.264/AVC, and H.265/HEVC, are applied to simulate the compression artifacts. Subjective rating scores in the form of mean opinion score (MOS) are given in the range of [0, 100], where a higher score means a better visual quality.

Our proposed method was run on a computer with a 3.60 GHz Intel Core i7 processor, 64 GB main memory, and Nvidia GeForce GTX 3090 graphics. We utilize three criteria to validate the performance of IQA models, including Pearson’s linear correlation coefficient (PLCC), Spearman’s rank-order correlation coefficient (SRCC), and root mean squared error (RMSE). For PLCC and SRCC, the higher the better, while for RMSE, the tendency is the opposite. For a perfect match between prediction scores and ground-truth subjective scores, we should have PLCC = SRCC = 1 and RMSE = 0. Before computing PLCC and RMSE, according to the recommendation of the video quality expert group (VQE) (Video Quality Experts Group, 2003), we apply a standard five-parameter logistic function to adjust the predicted scores to minimize the non-linearity of the subjective rating scores:


(16) $$\begin{array}{c} s=\alpha_{1}\,\left(\frac{1}{2}-\frac{1}{1+\exp\left(\alpha_{2}\,\left(p-\alpha_{3}\right)\right)}\right)+\alpha_{4}\,p+\alpha_{5} \end{array}$$

where *p* represents the predicted score and *s* denotes the mapped score. α_*1*_ to α_*5*_ are parameters for fitting this function.

Since our proposed method requires the training process to build the quality prediction model, it is necessary to describe the details regarding the training process. Specifically, we equally divide the whole dataset into five non-overlapping subsets with each subset containing 20% samples. Then, we apply the fivefold validation strategy to test the model performance. Specifically, the whole dataset is trained and tested five times, with four subsets as the training data and the remaining one subset as the testing data. After training and testing the whole dataset five times, we can get the predicted scores of all samples in the dataset. Finally, the PLCC, SRCC, and RMSE values are calculated between all the predicted scores and ground-truth subjective scores provided in each dataset.

### Single-dataset performance comparison

We compare the performance of our proposed S^3^DAVS model with eight representative NR-IQA models, including BRISQUE (Mittal et al., 2012), DIIVINE (Moorthy and Bovik, 2011), SSEQ (Liu et al., 2014), OG-IQA (Liu et al., 2016), NRSL (Li et al., 2016), SSP-BOIQA (Zheng et al., 2020), MC360IQA (Sun et al., 2019), and Zhou Y. et al. (2022) work. The former five models are traditional NR-IQA models that are developed for 2D natural images, while the latter three models are specifically designed for ODIs. In these three blind omnidirectional image quality assessments (BOIQA), MC360IQA (Sun et al., 2019) and Zhou Y. et al. (2022) work employ deep learning methods, while SSP-BOIQA (Zheng et al., 2020) focuses on segmenting ODI into three regions and extracting features with weights. Based on those training-based models, we re-trained the quality prediction models on each dataset with the same 5-fold validation strategy. Specifically, before testing the data, each dataset should generate three sub-datasets with three different scales respectively, and the reduction factor is 1/2. We fuse the features from sub-datasets into final feature vectors. Then, in the test session, a 5-fold cross-validation method is used to verify the performance of the model. The performance results in terms of PLCC, SRCC, and RMSE on the OIQA and CVIQD databases are shown in Tables 1, 2, respectively. We highlight the best performance model in each column. From these two tables, we have several observations. First, for both the OIQA and CVIQD databases, our proposed S^3^DAVS model, although inferior to some compared methods on some individual distortion types, presents the highest PLCC, SRCC, and RMSE values when considering the overall database. It means that our method is a good candidate for OIQA when the distortion type is unknown. Second, since the OIQA database contains more distorted types than the CVIQD database, the overall results on OIQA are commonly lower than those on the CVIQD database. According to the above experimental results, we could get the conclusion that the S^3^DAVS model owns the best performance among those models. Moreover, we can observe that the results of SSP-BOIQA (Zheng et al., 2020) which is designed for ODIs specifically are even worse than traditional 2D models. This is mainly due to the fact that SSP-BOIQA only focused on transforming the ERP image into bipolar and equatorial regions, ignoring the extraction of features from specific contents that are highly consistent with human perception.

**TABLE 1:** Performance comparison of the OIQA database.

Models	JPEG			JP2K			WN			BLUR			ALL		
	PLCC	SRCC	RMSE	PLCC	SRCC	RMSE	PLCC	SRCC	RMSE	PLCC	SRCC	RMSE	PLCC	SRCC	RMSE
BRISQUE (Mittal et al., 2012)	0.9401	0.8691	0.7605	0.8986	0.8447	0.9009	0.9663	0.9162	0.4664	0.9163	0.8529	0.7295	0.8972	0.8694	0.9358
DIIVINE (Moorthy and Bovik, 2011)	0.8963	0.8132	0.9307	0.9337	0.8970	0.7829	0.9649	0.9011	0.5029	0.9205	0.8392	0.7413	0.8793	0.8458	1.0127
SSEQ (Liu et al., 2014)	0.8905	0.8194	0.9945	0.8900	0.8500	0.9656	0.9589	0.9103	0.5200	0.9508	0.8989	0.6030	0.8970	0.8750	0.9240
OG-IQA (Liu et al., 2016)	**0.9552**	0.8912	**0.6845**	0.8759	0.8253	1.0224	0.9717	0.9206	**0.4262**	0.9473	0.9025	0.6005	0.9076	0.8954	0.8684
NRSL (Li et al., 2016)	0.9490	0.8834	0.7260	0.9538	0.8941	**0.5507**	0.9176	0.8691	0.8370	0.9258	0.8618	0.7074	0.8852	0.8537	0.9749
SSP-BOIQA (Zheng et al., 2020)	0.877	0.834	–	0.853	0.852	–	0.905	0.843	–	0.854	0.862	–	0.860	0.865	–
MC360IQA (Sun et al., 2019)	0.9015	0.8995	0.8234	0.8861	0.8779	1.3687	0.9195	0.9124	0.8234	0.8938	0.8892	1.3838	0.8953	0.8928	1.5052
Zhou Y. et al. (2022)	0.936	**0.940**	–	0.920	**0.934**	–	0.968	0.957	–	0.925	0.920	–	0.899	0.923	–
S^3^DAVS	0.9267	0.8872	0.8634	**0.9370**	0.9306	0.7717	**0.9725**	**0.9623**	0.4384	**0.9692**	**0.9662**	**0.4805**	**0.9405**	**0.9348**	**0.7183**

The best-performing NR metrics are highlighted in bold.

**TABLE 2:** Performance comparison on CVIQD database.

Models	JPEG			AVC			HEVC			ALL		
	PLCC	SRCC	RMSE	PLCC	SRCC	RMSE	PLCC	SRCC	RMSE	PLCC	SRCC	RMSE
BRISQUE (Mittal et al., 2012)	0.9519	0.9308	4.9825	0.8913	0.8559	5.6647	0.8979	0.8980	5.3367	0.9001	0.8814	6.2327
DIIVINE (Moorthy and Bovik, 2011)	0.9331	0.8710	6.0475	0.9024	0.8927	5.0618	0.9031	0.8530	5.6397	0.8988	0.9080	6.0260
SSEQ (Liu et al., 2014)	**0.9745**	**0.9527**	4.0731	0.9381	0.9180	4.2228	0.9115	0.9059	5.0884	0.9263	0.9134	5.2609
OG-IQA (Liu et al., 2016)	**0.9745**	0.9261	**3.6130**	0.8871	0.8852	5.7588	0.9030	0.9055	4.6374	0.9197	0.8969	5.3562
NRSL (Li et al., 2016)	0.9570	0.9056	5.1460	0.9145	0.8823	4.9565	0.9000	0.8981	4.8063	0.8850	0.8944	6.8612
SSP-BOIQA (Zheng et al., 2020)	0.915	0.853	6.847	0.885	0.861	7.042	0.854	0.841	6.302	0.890	0.856	6.941
MC360IQA (Sun et al., 2019)	0.9410	0.9230	5.8040	0.9320	0.9410	5.3570	0.9140	0.8990	4.8010	0.9390	0.9040	4.6060
Zhou Y. et al. (2022)	0.957	0.923	5.601	0.953	**0.949**	3.873	0.929	**0.914**	**4.525**	0.902	0.911	6.117
S^3^DAVS	0.9707	0.9302	3.8675	**0.9586**	0.9447	**3.3925**	**0.9367**	0.8802	4.5675	**0.9533**	**0.9426**	**4.1022**

The best-performing NR metrics are highlighted in bold.

### Statistical significance test

Besides the comparisons in terms of PLCC, SRCC, and RMSE, we employ that the *t*-test to demonstrate the superiority of our proposed S^3^DAVS model over other compared methods is significant. *T*-test could be divided into three types: one-sample *t*-test, independent samples *t*-test, and paired *t*-test, and is used to judge whether the performance difference between two models is significant or not. In this section, we first get 100 results of indicators and select the PLCC as the sample data, and then use the independent samples *t*-test method to calculate the final significance criteria. The formula can be written as:


(17) $$\begin{array}{c} t=\frac{m_{A}-m_{B}}{\sqrt{\frac{S_{A}^{2}}{n_{A}}+\frac{S_{B}^{2}}{n_{B}}}} \end{array}$$

**FIGURE 5:**
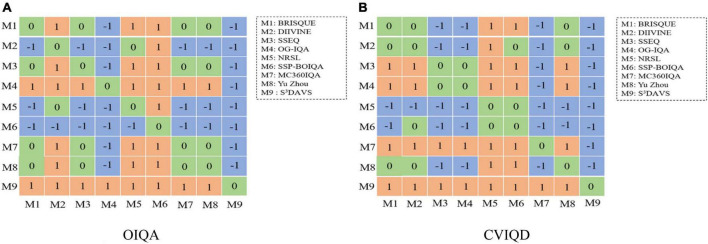
Statistical significance comparison by the t-test between our proposed method and other methods. **(A,B)** Depict the results on the OIQA and CVIQD databases, respectively.

For each model, *m* represents the means of samples, *S*^2^ represents the variance of samples, and *n* is the number of samples. *t* illustrates the significant difference between the two compared models. In Figure 5, the blue block indicates that the model at a row is worse than model in the column and is labeled as “-1.” The green block indicates that there are no obvious differences between the models in row and column and is labeled as “0.” The orange block means the model at row is worse than that in column, and is labeled as “1.” According to the data distribution of each row in the statistical significance figure, the higher the number of “1,” the better the algorithm. It can be observed that our proposed model is significantly better than all the others.

### Ablation study

The proposed method mainly focuses on extracting statistical features in the spatiotemporal domain and analyzing the viewport content in a way that is consistent with human viewing behavior. According to the aforementioned feature extractor, we know that our method mainly depends on the spatiotemporal MSCN (ST-MSCN) and the spatiotemporal Gabor filter (ST-Gabor) to obtain feature vectors. To figure out the performance of each module, we test the performance by considering each single feature set, i.e., only using the feature set from the ST-MSCN or the ST-Gabor filter. The results are shown in Tables 3, 4, respectively. By comparing the results in these two tables, we can easily find that the features from the ST-Gabor play a dominant role, while the spatiotemporal MSCN features play an auxiliary role in our S^3^DAVS. Overall, the performance will be further improved by jointly considering the spatiotemporal statistical features of the ST-Gabor and the ST-MSCN.

**TABLE 3:** Performance comparison of the OIQA database with each single feature set.

Ablation models	PLCC	SRCC	RMSE
ST-MSCN	0.6303	0.5901	1.6346
ST-Gabor	0.9259	0.9213	0.8130
ST-MSCN + ST-Gabor	**0.9405**	**0.9348**	**0.7183**

The best-performing results are highlighted in bold.

**TABLE 4:** Performance comparison on the CVIQD database with each single feature set.

Ablation models	PLCC	SRCC	RMSE
ST-MSCN	0.6529	0.6091	10.6939
ST-Gabor	0.9223	0.9041	5.5126
ST-MSCN + ST-Gabor	**0.9533**	**0.9426**	**4.1022**

The best-performing results are highlighted in bold.

### Cross-dataset performance comparison

To test the generalization ability of an image quality metric, cross-database validation is necessary. Given two databases, we use one database for training and another one for testing. Because the OIQA database contains more distorted types, such as Gaussian noise and Gaussian blur, whereas the CVIQD database only contains compression distortion, we only tested JPEG and JP2K in the OIQA database, and the results are shown in Table 5. As shown in the table, the results on the CVIQD database are generally higher than the results on the OIQA database. The reason is that the model trained on the OIQA database learns more feature types and could discriminate the distortion types in the CVIQD database more accurately. Overall, in comparison to other existing methods, our proposed model has a better generalization capability.

**TABLE 5:** Performance results of cross-database validation.

Models	Train OIQA/Test CVIQD			Train CVIQD/Test OIQA		
	PLCC	SRCC	RMSE	PLCC	SRCC	RMSE
BMPRI (Min et al., 2018)	0.4904	0.2417	12.1862	0.7595	0.7205	1.3249
CEIQ (Yan et al., 2019)	0.6953	0.5470	9.9767	0.5012	0.4860	1.7856
BRISQUE (Mittal et al., 2012)	0.6166	0.5503	11.1772	0.4950	0.4054	1.8217
DIIVINE (Moorthy and Bovik, 2011)	0.5658	0.4114	11.4963	0.4454	0.3575	1.8904
SSEQ (Liu et al., 2014)	0.6175	0.6113	10.8955	0.4927	0.4568	1.7922
NRSL (Li et al., 2016)	0.6884	0.6199	10.4646	0.3651	0.2648	1.9431
OG-IQA (Liu et al., 2016)	0.6963	0.6392	10.1059	0.5154	0.5299	1.8076
SSP-BOIQA (Zheng et al., 2020)	0.726	0.705	9.588	0.627	0.601	–
MC360IQA (Sun et al., 2019)	0.8230	0.8140	7.8110	0.6816	0.5238	1.5471
Zhou Y. et al. (2022)	**0.847**	**0.825**	**7.721**	0.735	0.741	–
S^3^DAVS	0.8358	0.8125	7.9331	**0.7817**	**0.6859**	**1.3938**

The best-performing results are highlighted in bold.

## Conclusion

This article has presented a novel no-reference (NR) ODI quality evaluation method based on the construction of Dynamically Attentive Viewport Sequence (DAVS) from ODIs and the extraction of Quality-Aware Features (QAFs) from DAVS. The construction of DAVS aims to build a sequence of viewports that are likely to be explored by viewers based on the prediction of visual scanpath when viewers are freely exploring the ODI within the exploration time *via* HMD. A DAVS that contains only global motion can then be obtained by sampling a series of viewports from the ODI along the predicted visual scanpath. The subsequent quality evaluation of ODIs is performed merely based on the DAVS. The extraction of QAFs aims to obtain effective feature representations that are highly discriminative in terms of perceived distortion and visual quality. Finally, a regression model is built to map the extracted QAFs to a single predicted quality score. Experimental results on two datasets demonstrate that the proposed method is able to deliver state-of-the-art performance. However, its shortcomings are also obvious. Although this method captures effective information to analyze the feature of ODI and predict the quality, it is time-consuming and computationally complex, and it needs to be improved. Taking the OIQA database as an example, there are 320 ODIs in this database, and the smallest resolution is 11,332 × 5,666. The test result shows it takes about 1.983 min for an image to complete all the steps. Besides, the scanpath can only present the movements of human eyes, but the movements of the head are not clear. We could explore these directions in our future work.

## Data availability statement

The original contributions presented in this study are included in the article/supplementary material, further inquiries can be directed to the corresponding author.

## Author contributions

YW and HL contributed to conception and design of the study and wrote the first draft of the manuscript. YW wrote the software codes of the algorithm and conducted experiments. HL and QJ performed the statistical analysis. All authors contributed to manuscript revision, read, and approved the submitted version.
